# Application of Affective Touch in Patients With Advanced Cancer: Effects on Existential Distress, Pain, and Quality of Life

**DOI:** 10.1097/jnr.0000000000000735

**Published:** 2026-03-25

**Authors:** Hong-Tao ZHENG, Wei WANG, Ju-Ling XU, Hua-Fang LI, Jing ZHANG, Ying WU

**Affiliations:** 1 The First People’s Hospital of Yongkang, Affiliated to Hangzhou Medical College, Jinhua, China.; 2 School of Medicine, Huzhou University, Huzhou, China

**Keywords:** affective touch, Swanson’s caring theory, advanced cancer, existential distress, quality of life

## Abstract

**Background:**

Patients with advanced cancer generally experience adverse physical and mental burdens. As a nondrug approach, affective touch provides support for oncology nurses in helping patients with advanced cancer overcome these burdens.

**Purpose:**

This study was designed to implement an affective touch intervention for patients with advanced cancer to explore its effects on existential distress, pain, and quality of life in this vulnerable patient population.

**Methods:**

In this randomized controlled trial, 83 patients with advanced cancer were allocated randomly to either the experimental or control group. The experimental group received the affective touch intervention once a day for 2 weeks. General data were collected from all of the participants before the intervention. Existential Distress Scale and Functional Assessment of Cancer Therapy-General scores were collected both before and 2 weeks after the intervention. Average pain levels on the Brief Pain Inventory were collected before and at weeks 1 and 2 of the intervention. Current pain levels were collected daily after the intervention.

**Results:**

After completing the 2-week intervention, the total scores for the control and experimental groups were, respectively, 8.15 ± 2.23 and 6.60 ± 2.75 (*p* < .05) for existential distress and 50.24 ± 12.35 and 57.24 ± 14.26 (<.001) for quality of life, showing a significant improvement effect for the intervention. Affective touch was shown to have an immediate ameliorative effect on pain after the daily intervention but was not shown to be significantly effective in providing long-term pain relief at either 1 or 2 weeks after the intervention.

**Conclusions/implications for Practice:**

Affective touch, as a simple, noninvasive, and cost-effective approach implemented alongside routine nursing care, may improve existential distress, immediate pain, and quality of life in patients with advanced cancer. The affective touch intervention is worth promoting and applying in clinical settings. Further efforts by senior practice nurses are needed to incorporate this intervention into medical oncology practice.

## Introduction

### Background

According to the latest global cancer burden data released by the World Health Organization’s International Agency for Research on Cancer (IARC; Sung et al., 2021), 19.3 million new cancer cases and 10 million cancer deaths were reported worldwide in 2020. Cancer is a major public health problem and a significant social burden worldwide. Patients with cancer experience both acute and chronic symptoms caused by the underlying disease or treatment (Panigrahi et al., 2022). The development of cancer, with symptoms such as dyspnea, pain, appetite loss, general fatigue, and sleep disturbance, can significantly reduce the quality of life of patients with cancer (Biswas & Acharyya 2020; Zhang et al., 2022). In addition, existential distress, an unpleasant psychological experience that occurs when the body cannot bear current living conditions amidst long-term pressure, is commonly observed in patients with advanced cancer and other life-threatening diseases (Xu et al., 2024). The concept of existential distress includes five core attributes: lack of meaning, loss of autonomy, loss of dignity, hopelessness, and death anxiety. Existential distress is a key factor causing poor quality of life, a poor emotional state, demoralization, and even suicide (Chen et al., 2022). In the current clinical cancer treatment environment, when curative treatment is no longer beneficial, reducing the physical and mental symptoms experienced by patients with cancer is a more realistic and feasible focus (Dingley et al., 2021). Under this scenario, effective intervention measures are particularly important to implement for patients with advanced cancer to minimize the effect of psychological pressure on symptoms, reduce their physical and mental burden, and allow them to maintain a higher quality of life through the remainder of their lives.

Touch, an important means through which humans receive information about the outside world, helps regulate social perception in various ways that affect people’s thoughts and feelings and shape the interactive atmosphere (Schirmer et al., 2023). Touch may be classified into two dimensions: sense–discrimination dimension (mediated by A-β fiber of perimedullary nerve fiber) and motivation–emotion dimension (tactile conduction without perimedullary nerve fiber C). The motivation–emotion dimension has biophysical, electrophysiological, neurobiological, and anatomical properties that drive a temporarily delayed emotional-somatic system (McGlone et al., 2007). Touch in the emotional dimension refers to tactile processing with a pleasurable or emotional component (Chen et al., 2020). This affective tactile system is projected through brain regions associated with emotional processing and social connection (Olausson et al., 2002), causing arousal and titer fluctuations related to homeostasis needs, in addition to the comfort, sedation, and analgesic benefits of touch itself (Habig et al., 2017), which may also be influenced by the corresponding regulation of the autonomic nervous system and endocrine pathways (Fotopoulou et al., 2022; Meijer et al., 2022). Some scholars have defined affective touch as touching behavior that is used to transmit information or express feelings such as love, care, and appreciation (Yang & Zhu, 2022). In the field of medicine, affective touch refers to the use of empathy, empathy respect, and other skills by medical staff to provide emotional comfort to patients and reduce their loneliness and uncertainty regarding a disease (Schimidt & da Silva, 2013). In this context, touch is performed by observing, perceiving, and analyzing the needs of the other party and determining the appropriate caring action mode. It primarily includes physical touch (caressing, massage, or acupressure) and spiritual touch (emotional communication, spiritual support, interpersonal coordination, and problem solving; Xiuxia et al., 2016). Therefore, affective touch is based on humanistic care that uses empathy, respect, and other modes to provide physical and spiritual touch and comfort, convey care and support to the touched and to reduce existential distress.

In Smith and Reed (2023), 192 patients with advanced cancer were touched once a week for 30 min/time, with their pain, mood and quality of life surveyed at the end of the touch and at 1 week after the touch. The results showed touch immediately and consistently reduced pain and improved both mood and quality of life. After conducting structured interviews with the patients, the three themes of perception, reflection, and connection were extracted and described, and it was concluded that touch was a comfortable and relaxing experience for the patients that made them self-focus, caused them to think about life, and helped them regain meaning in life. Similarly, 35 patients with advanced cancer were provided touch therapy addressing the head, chest, hands, legs, and feet at an assisted care center (Weze et al., 2004), one time per week for 40 min per session over a 4-week period. The patient lies comfortably on the treatment bed, and during the touch, the touch therapist in emotional conversation with the patient and listens sympathetically, noting physical, mental, emotional, and spiritual changes since the last treatment. Despite the simple, repetitive touch pattern employed, the results show patients significantly improved in six symptom areas (e.g., stress, pain, fear) and experienced improved quality of life. In 2017, Zhou et al. (2017) implemented touch interventions, including physical and mental touch, for patients with breast cancer undergoing chemotherapy. Body touch was based on acupoint touch, while mental touch was a “soul touch” approach based on observation and emotively communicating with patients, with the first intervention session given 1 hour before the first chemotherapy session and continuing until the end of the chemotherapy course for 1 time/day, 0.5 ~ 1 hour/time. The results showed that touch effectively relieved anticipatory nausea and vomiting symptoms and improved negative emotions.

In light of the above, evidence supports using affective touch as an emotional regulation channel between people can help solve the emotional problems and physical discomfort experienced by cancer patients. The purpose of this study was to measure the reduction effect of affective touch on existential distress and immediate pain in patients with advanced cancer and the effect on their quality of life, with the goal of identifying a more effective intervention to reduce physical and mental pain and improve quality of life in this vulnerable patient population.

## Methods

### Design

A randomized controlled design was used, and participants were recruited from among patients with advanced cancer receiving treatment in an oncology department. A digital custodian not directly involved in screening, patient recruitment, or clinical nursing used SPSS version 25 to generate random numbers, which were then randomized into two groups. The newly generated results were the randomized grouping of each participant, with “1” representing the control group and “2” representing the experimental group. Sequentially numbered, opaque, sealed envelopes were used to conceal the sequence until the interventions were assigned at the oncology department. Then, these envelopes were numbered and distributed to participants by a research assistant based on their enrolment sequence. The intervention period was from January to December 2023. The control group received conventional medical care only, while the experimental group received the affective touch nursing intervention in addition to conventional medical care. Baseline and outcome data were collected before and after the intervention, respectively.

### Setting and Participants

**Figure 1:**
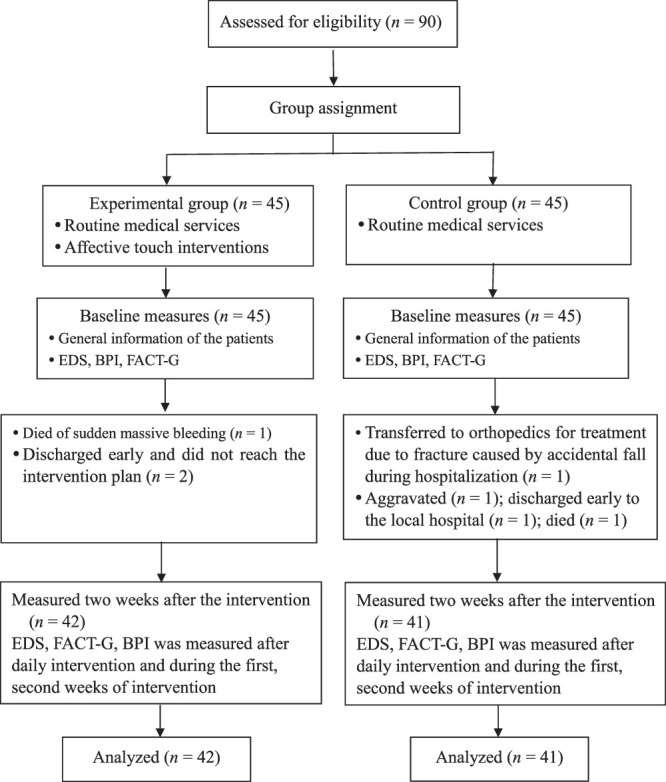
Procedure for Recruiting Study Participants, Intervention, and Measurements Flowchart *Note.* BPI = Brief Pain Inventory; EDS = Existential Distress Scale; FACT-G = Functional Assessment of Cancer Therapy-General.

Patients who met the following inclusion criteria were invited to participate: (a) diagnosed with stage III or IV cancer based on pathology, imaging, and clinical examination; (b) at least 18 years of age and aware of their disease diagnosis, condition, and treatment; (c) good cognition and good communication and comprehension skills; (d) absence of small fiber neuropathy; and (e) aware, along with their main caregivers, of the study’s purpose and methods and willing to participate voluntarily and sign informed consent. The exclusion criteria were (a) platelet count below 100*109/L, (b) thrombosis, trauma, and skin diseases in both upper limbs; (c) prior mental illness diagnosis, and (d) participation in other research groups. A flowchart of the procedures used in recruiting participants, performing the intervention, and collecting measurements is shown in Figure 1.

### Ethical Considerations

This study was approved by the ethics review committee of a hospital (YKRM2023- LS030). The study objectives and the rights and risks of participation were explained to the participants, who were provided the opportunity to ask any questions. Written informed consent was obtained from all of the participants.

### Measurements

The researcher prepared a general information questionnaire for collecting demographic information from the participants before the intervention, which included gender, age, educational level, marital status, religious affiliation, primary caregiver, type of medical expense payment, clinical diagnosis, duration of disease, cancer stage, metastasis status, treatment mode, and pain score.

The Existential Distress Scale (EDS), designed by Canadian scholars Lo et al. specifically for advanced cancer patients, includes 10 items in the following three dimensions: meaninglessness (Items 8–10), loneliness (Items 1–3), and worthlessness (Items 4–7). The scale has good psychometric characteristics, with a Cronbach’s alpha of .86 (Lo et al., 2017). Higher total scores are associated with worse existential distress. Xiao et al. (2022) subsequently translated and localized the EDS for use in China and confirmed its reliability and validity. The Chinese version of the EDS, with a Cronbach’s alpha of .892 was used in this study.

The Brief Pain Inventory (BPI), developed by the Pain Research Group of the World Health Organization Cancer Care Symptom Assessment Collaborating Centre, is distinguished into two dimensions: pain intensity (sensory dimension) and pain interference with patients’ lives (response dimension; Cleeland & Ryan, 1994). It is commonly used to evaluate the degree of pain in patients with cancer. The sensory dimension includes the pain location in the past 24 hours, average degree of pain, highest degree of pain, lowest degree of pain, current degree of pain, and pain relief after pain management in the past 24 hours. Pain severity is rated on a scale of 0 to 10, with 0 representing *no pain at all* and 10 representing *the most pain*. Prior internal consistency reliability analysis has revealed good internal consistency for the BPI (Cleeland & Ryan, 1994). Wang et al. (1996) subsequently translated this scale into Chinese. In this study, the current and average pain levels in the Chinese version of the Brief Pain Inventory (BPI-C) were respectively used to assess the immediate and sustained effects of the intervention, with a Cronbach’s alpha of .894.

The Functional Assessment of Cancer Therapy (FACT), developed by Cella et al. (2023), includes the generic scale FACT-G and several cancer-specific modules. FACT-G plays a crucial role in the entire system and may be used either alone or in combination with specific modules to assess the quality of life experienced by patients with various cancers. Internal consistency reliability analysis has revealed the scale to have good internal consistency (Cronbach’s alpha > .82 for all dimensions). FACT-G consists of 27 items across four domains: physiological status (7 items), social/family status (7 items), emotional status (6 items), and functional status (7 items). Each item is rated on a 5-point scale scored as follows: *not at all* (0), *a little* (1), *some* (2), *quite* (3), and *very* (4). Positive entries are scored from 0 to 4, while reverse entries are scored from 4 to 0, with higher scores associated with better quality of life (Cella et al., 2023). The FACT-G Chinese version of the fourth (latest) edition of FACT was used in this study, with Cronbach’s alpha values >.8 for all dimensions.

### Intervention

The control group received only routine medical care, which included an introduction to environmental settings, basic care, symptom control, psychosocial assessment, physical symptom monitoring, comfort care, mental and social support, and guidance for daily living. In addition to routine medical care, the experimental group received an affective touch intervention that included both physical and psychological touch. The intervention plan used in this study was framed around the hand touch program developed by Snyder et al. (1995), Swanson's care theory, and the domestic care plan for cancer patients based on Swanson's care theory (Gilbert & Lillekroken, 2023). The final version was developed after repeated discussions and modifications by the expert group (Table 1) and was implemented by oncology nurses who had received 1 month of professional training under the guidance of a physiotherapist. Due to medical insurance restrictions in China, hospitalized patients generally stay for 2 weeks. The intervention was administered for 30 min beginning at 16:00 every day for 2 weeks.

**Table 1:** Contents of the Affective Touch Intervention

Theme	Intervention Content	Intervention Measure	Remark
Full knowledge	Psychic touch and hand touch	Question: ① Tell me about the most urgent problem you need to solve today? ② Tell me the one thing that you are most concerned about today? ③ Tell me about what you and your family did today. ④ Tell me about one thing that made you happy today? ⑤ What regrets do you have today? The implementers listen patiently to the demands of the patients, and understand the psychological experience of the patients from the body language or communication with them, so as to obtain effective information.	Communication takes place simultaneously with hand touch
Full company	Hand touch	① Short/medium length straight strokes from wrist to fingertips using moderate pressure,1 min. ② Back of hand: 1 min. A: Large half-cimular stretching strokes from center to side of hand using moderate pressure. B: Small circular strokes over entire back of hand, using light pressure (making little O’s with thumb). C: Feather like straight strokes from wrist to fingertips using very light pressure. ③ Palm of hand: 1 min. A: Short/medium length straight strokes from wrist to fingertips using moderate pressure. B: Gentle milking/lifting of tissue of entire palm of hand using moderate pressure. C: Small circular strokes over entire palm of hand using moderate pressure (making little O’s). D: Large half-circular stretching strokes from center of palm to sides using moderate pressure. ④ Finger:1 min A. Gentle squeezing of fingers from base to tip on sides and top/bottom using light pressure B. Gentle circular range of motion of each finger followed by a gentle squeeze of the nail bed. ⑤ Completion of hand: 1 min Lay resident’s hand on yours and cover it with your other hand. Gently draw your top hand toward you several times. Turn resident’s hand over and gently draw the other hand toward you several times.	Repeat with the other hand
Offer help	Psychic touch and hand touch	Question: ① What can I do for you? Answer patients’ questions, provide appropriate guidance and help; Maintain the dignity of patients, let patients feel the comfort of love and belonging.	Do the same for hand touch
Full enabling	Psychic touch	Question: ① Tell me about your short-term goals? Encourage patients to take care of themselves and their families and develop patients’ confidence; Emphasize that patients accept themselves, and then guide self-realization; Instruct patients who wish to master hand touch and encourage them to learn and try.	Assist in achieving goals during the intervention cycle
Maintain faith	Psychic touch	Further provide guidance to patients who are willing to master hand touch, including touch. Assist in achieving goals during the intervention cycle technique, strength, location, etc. Accepting the expression of patients’ negative feelings and telling examples of successful treatment, patients are encouraged to maintain a hopeful attitude, and patients are helped to obtain and maintain hope and regain confidence in exploring the meaning of life.	

To verify the feasibility of the scheme design and better identify practical issues, we selected additional patients representing 10% of the sample size who met the inclusion and exclusion criteria. These 10 advanced cancer patients (five in the control group and five in the experimental group) participated in pre-experiments conducted before the formal intervention. The results showed that after 2 weeks of the intervention, the pain relief effect on these patients was not significant. Thus, after discussion, daily pain assessment was added immediately after the intervention to observe the immediate effect of the intervention, and the average pain level was evaluated weekly to observe the sustained effects.

### Data Collection

In this study, general patient data were collected before the intervention; EDS and FACT-G scores were collected before and 2 weeks after the intervention; average pain levels using the BPI were collected before the intervention and at weeks 1 and 2; and current pain levels were collected daily after the intervention.

### Data Analysis

Data were analyzed using IBM SPSS Statistics Version 25.0 (IBM Corp., Armonk, NY, USA). Descriptive statistics were utilized to compare the demographic and baseline characteristics of the two groups, with χ^2^ and Fisher’s exact tests used for categorical variables and *t* tests used for continuous variables. Two independent samples *t* test and a repeated-measure analysis of variance were used to examine the between-group difference before and after each intervention session. The significance level for all tests was set as *p* < .05.

## Results

A total of 90 patients with advanced cancer were initially included in the study as participants. After the withdrawal of seven participants, the control and experimental groups, respectively, contained 41 and 42 participants each. Thus, 83 participants completed the study protocol and were included in the statistical analysis. No significant differences between the two groups were found in terms of disease characteristics, although a between-group difference in gender makeup was identified in the general characteristics (Table 2).

**Table 2:** Between-Group Comparison of General Data

Variable	Control Group (*n* = 41)	Experimental Group (*n* = 42)	*t*/χ²	*p*
	*n* (%)	*n* (%)		
Age (years; *M* and *SD*)	65.32±13.17	62.26±12.06	1.103	.273 ^a^
Gender			4.490	.047 ^b^
Male	28 (68.3)	19 (45.2)		
Female	13 (31.7)	23 (54.8)		
Education			3.134	.546 ^c^
Illiterate	3 (7.3)	6 (14.3)		
Primary school	16 (39.0)	19 (45.2)		
Junior high school	16 (39.0)	12 (28.6)		
High school and technical secondary school	6 (14.6)	4 (9.5)		
Junior college	0 (0.0)	1 (2.4)		
Bachelor's degree or above	0 (0.0)	0 (0.0)		
Marital status			2.065	.514 ^c^
Unmarried	0 (0.0)	0 (0.0)		
Married	38 (92.7)	37 (88.1)		
Divorced	2 (4.9)	1 (2.4)		
Death of a spouse	1 (2.4)	4 (9.5)		
Religious belief			0.000	1.000 ^c^
Buddhism	1 (2.4)	1 (2.4)		
Christianity	0 (0.0)	0 (0.0)		
Taoism	0 (0.0)	0 (0.0)		
No	40 (97.6)	41 (97.6)		
Medical payment method			0.398	1.000 ^c^
Self-financing	0 (0.0)	0 (0.0)		
Medical insurance for urban residents	6 (14.6)	5 (11.9)		
Medical insurance for urban workers	2 (4.9)	3(7.1)		
New rural cooperative medical system	33 (80.5)	34 (81.0)		
Monthly income (RMB)			2.379	.719 ^c^
<1,000	13 (31.7)	17 (40.5)		
1,001–2,000	15 (36.6)	12 (28.6)		
2,001–3,000	6 (14.6)	9 (21.4)		
3,001–4,000	4 (9.8)	2 (4.8)		
>4,000	3 (7.3)	2 (4.8)		
Primary caregiver			1.070	1.000 ^c^
Parents	0 (0.0)	1 (2.4)		
Spouse	26 (63.4)	26 (61.9)		
Sons and daughters	13 (31.7)	13 (31.0)		
Other	2 (4.9)	2 (4.8)		
Clinical diagnosis			2.100	.925 ^c^
Lung cancer	19 (46.3)	18 (42.9)		
Rectal cancer	5 (12.2)	5 (11.9)		
Gastric cancer	5 (12.2)	7 (16.7)		
Carcinoma of bladder	1 (2.4)	0 (0.0)		
Ovarian cancer	2 (4.9)	4 (9.5)		
Other	9 (22.0)	8 (19.0)		
Duration of illness (months; *M* and *SD*)	20.68±19.65	28.21±32.89	−1.263	.210 ^a^
Metastasis or not			1.867	.200 ^b^
Yes	29 (70.7)	35 (83.3)		
No	12 (29.3)	7 (16.7)		
Tumor staging			1.029	.350 ^b^
Phase III	7 (17.1)	4 (9.5)		
Phase IV	34 (82.9)	38 (90.5)		
Treatment mode			2.249	.719 ^c^
Palliative care	15 (36.6)	15 (35.7)		
Surgical treatment	13 (31.7)	16 (38.1)		
Chemotherapy	5 (12.2)	2 (4.8)		
Combination therapy	6 (14.6)	8 (19.0)		
Others	2 (4.9)	1 (2.4)		
Pain (*M* and *SD*)	3.05±1.02	3.07±1.09	−0.097	.923 ^a^

^a^ *t* test. ^b^ χ^2^ test. ^c^ Fisher's exact probability method.

As shown in Table 3, no significant differences between the two groups were found in terms of existential distress and quality of life before the intervention. After the intervention, the existential distress score of the experimental group was 6.60 (*SD* = 2.75), and that of the control group was 8.15 (*SD* = 2.23), revealing a statistically significant between-group difference in existential distress (*p* < .05; Table 3).

**Table 3:** Psychological Outcomes Before and After the Affective Touch Intervention (*N* = 83)

Evaluation index/Evaluation Time	Experimental Group(*n* = 42)	Control Group(*n* = 41)	Difference / 95% CI	*t*	*p*
	*M* ± *SD*	*M* ± *SD*			
Existential distress			1.55 [0.46, 2.66]		
Preintervention	8.98±3.91	9.46±2.77		0.654	.515
Postintervention	6.60±2.75	8.15±2.23		2.818	.006
Physiological			1.61[3.95, 7.22]		
Preintervention	12.64±5.46	12.66±5.41		0.013	.990
Postintervention	15.88±5.35	14.27±5.34		1.374	.173
Social/family			1.96 [0.33, 3.58]		
Preintervention	11.64±4.10	11.00±3.27		0.788	.433
Postintervention	14.81±4.06	12.85±3.43		2.392	.019
Emotional			3.04 [1.45, 4.62]		
Preintervention	12.45±3.84	13.24±4.10		0.908	.367
Postintervention	19.55±3.47	16.51±3.77		3.817	<.001
Functional			0.49 [1.34, 2.32]		
Preintervention	5.02±3.81	5.44±4.15		0.475	.636
Postintervention	7.00±4.17	6.51±4.21		0.530	.597
FACT-G total			6.99 [1.16, 12.83]		
Preintervention	41.40±14.08	41.93±13.13		0.175	.862
Postintervention	57.24±14.26	50.24±12.35		2.386	.019

*Note.* FACT-G = Functional Assessment of Cancer Therapy-General; CI = confidence interval.

The social/family status and emotional status dimension scores and FACT-G total scores of the two groups showed a statistically significant difference (*p* < .05), while a comparison of the physiological status and functional status dimensions found no statistically significant difference (*p* > .05; Table 3).

**Figure 2:**
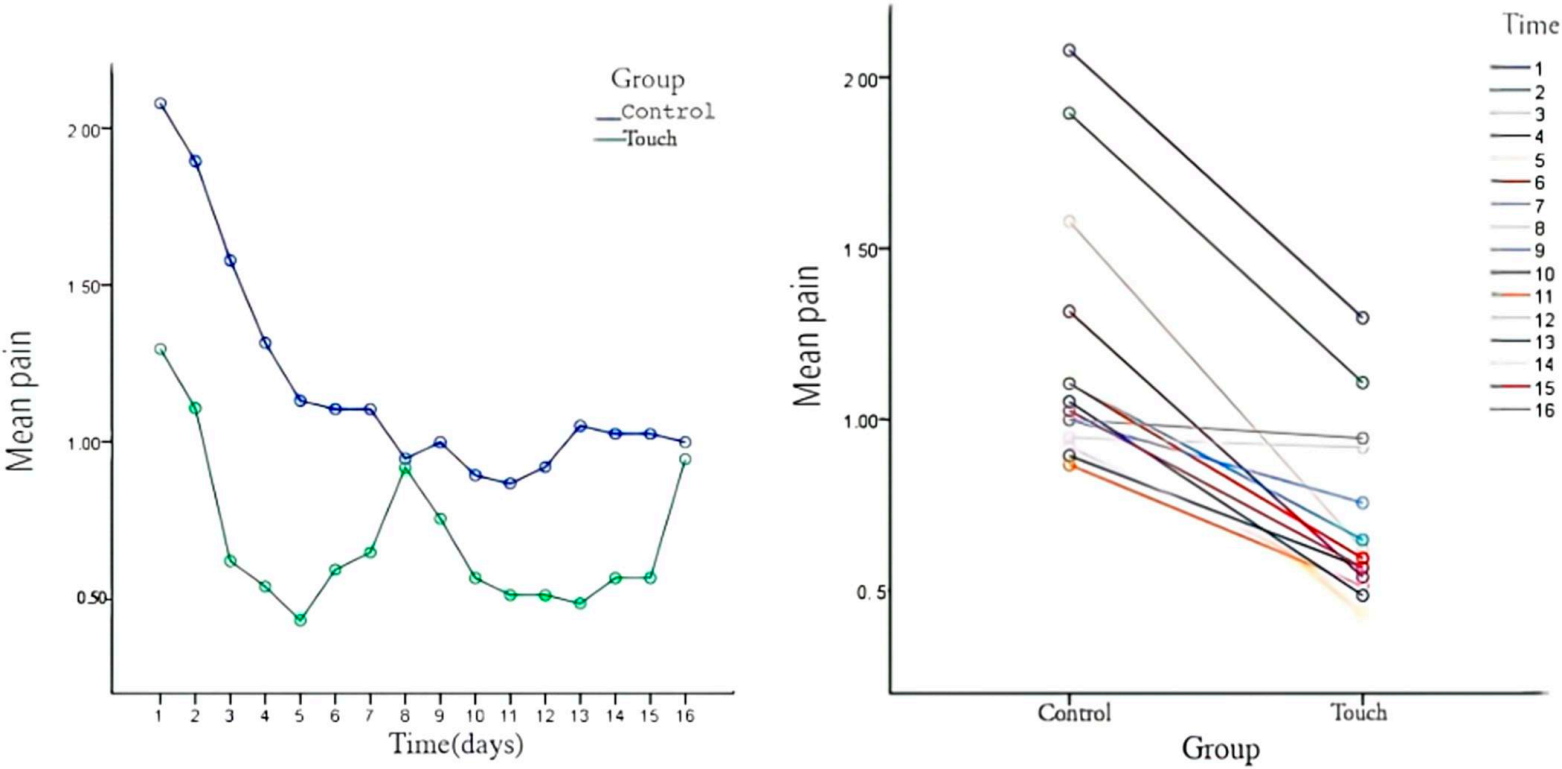
Between-Group Comparisons of Self-Perceived Pain at Each Time Point

In terms of pain, before the intervention, no significant differences were found in BPI between the two groups (*p* > .05). However, after the intervention, a repeated-measure analysis of variance showed statistically significant differences in BPI between groups (*F*-group = 87.906, *p* < .001) which were all affected by time factors, with 16 statistically significant differences at different times (*F*-time = 25.484, *p* < .001). The duration and intergroup interaction of BPI between the two groups (*F*-interaction) was 4.251 (*p* < .001). Furthermore, simple effect tests identified significant differences in the BPI score between the two groups after daily interventions. Pain relief in the experimental group was significantly better than that in the control group, indicating the affective touch intervention improves immediate pain perception in patients with advanced cancer. However, after weeks 1 and 2 of the intervention, the simple effect score of the BPI for both groups was not significant, indicating affective touch had no significant lasting effect on pain relief. The BPI score decreased significantly in the experimental group after the daily intervention and showed no significant decreasing trend over time (week 1 and week 2). However, in the control group, the BPI score decreased significantly during the postintervention period, with the range of change at each subsequent time point showing a stable trend (Figure 2).

## Discussion

A gender difference between the two groups was found in this study. In terms of all participants, males comprised over half of the sample (47; 56.6%), while the 36 females accounted for 43.4% of the sample. Reasons for this may include lung cancer being the most prevalent form of cancer in the sample; males having a higher propensity to engage in cancer-related behaviors such as smoking, drinking, unhealthy eating, and other unhealthy lifestyle behaviors (Qiu et al., 2022); and males being less likely to take cancer screening tests. Most of the participants had a relatively low level of education, with most (86.7%) educated to the junior high school level or less. Also, nearly all (97.6%) self-described as having no religious affiliation. Most had secondary metastasis (77.1%), and caregivers were mostly spouses (62.7%) and children (31.3%). The average disease duration was (24.49 ± 2.99) months, reflecting the targeted recruitment of advanced cancer patients in this study. With the increase in treatment avenues and implementation of national primary prevention and screening work, the 5-year survival rate of cancer in China has significantly improved in recent decades (Maomao et al., 2022).

Existential distress is very common among cancer patients (Nanni et al., 2018; Ueland et al., 2021), with patients suffering from self-crisis at the physical, emotional, and/or spiritual level, potentially leading to a sense of worthlessness, loneliness, and meaninglessness in life.

In this study, the EDS was used to evaluate survival distress in 83 patients with advanced cancer. After 2 weeks of the intervention, existential distress in the experimental group was significantly improved over that of the control group (*p* < .05). There were several main reasons for conducting this analysis. Combined with comprehensive hand touch therapy, care and help are provided from the physical level, while spiritual touch provides emotional support from the psychological level, making patients feel love and belonging, thus alleviating loneliness and enhancing their sense of self-worth. In undergoing the process of “maintaining faith” in Swanson’s caring theory, patients regain their self-confidence in exploring the meaning of life by maintaining their hope and optimism, thus alleviating their sense of meaninglessness. (loneliness and enhancing their sense of self-worth. In undergoing the process of “maintaining faith” in Swanson’s caring theory, patients regain their self-confidence in exploring the meaning of life by maintaining their hope and optimism, thus alleviating their sense of meaninglessness. (loneliness and enhancing their sense of self-worth. In undergoing the process of “maintaining faith” in Swanson’s caring theory, patients regain their self-confidence in exploring the meaning of life by maintaining their hope and optimism, thus alleviating their sense of meaninglessness. (loneliness and enhancing their sense of self-worth. In undergoing the process of “maintaining faith” in Swanson’s caring theory, patients regain their self-confidence in exploring the meaning of life by maintaining their hope and optimism, thus alleviating their sense of meaninglessness. (Van den Berg et al., 2006) implemented a touch intervention with 57 cancer patients and evaluated the effects on their pain levels and quality of life. The investigators intervened with each patient for at least five 45-min touch sessions. The location of the touch depends on patient needs and usually involves the back, legs, feet, abdomen, neck, and shoulders, with conversations on a psychological level during the touch. The findings showed touch intervention improved the patients’ cognitive and social functioning, reduced distress levels, and improved overall quality of life. Smith et al. (2023) found that touch can bring comfort and relaxation to patients with advanced cancer, allowing them to focus more on their own experiences and rediscover the meaning of life.

In this study, BPI was used to evaluate the pain symptoms of 83 patients with advanced cancer, with 96% of the patients reporting pain before intervention. After the intervention, BPI was evaluated at 16 time points, with the results showing affective touch interventions to be effective in improving immediate pain issues, but not provide significant persistent effects. The results of this study are similar to those of Kutner (Kutner et al., 2008), who administered six 30-min touch sessions to patients with advanced cancer over a 2-week period and concluded that touch was associated with a significant improvement in immediate pain but did not have a statistically significant effect on continuous measurements. Cadet et al. (2022) conducted touch interventions on cancer patients, with results showing that touch helped manage both physical and emotional symptoms, relieving pain in 90% of participants. The participants in this study were all in the advanced stage of cancer, and their use of analgesic drugs did not change significantly during the study period. Therefore, the pain changes observed may feasibly be attributed to the influence of affective touch. Affective touch triggers the secretion of endogenous hormone levels through the reward area of the brain. These endogenous hormones can regulate psychological stress (Pearce et al., 2017) and reduce heart rate and pain levels (Ditzen et al., 2007). Several potential analgesic mechanisms for touch and massage have been suggested (e.g., Kocot-Kępska et al., 2021), including (a) touch-activated peripheral nerve endings inhibit the transmission of pain stimuli; (b) reduced anxiety and stress levels in cancer patients reduce perceived pain levels; and (c) relieving pain as a distraction or interruption. In this study, BPI scores were self-reported by the participants. The spiritual touch based on Swanson’s caring theory can make patients feel more cared for and supported psychologically during the intervention, which may alleviate stress. Concurrently, hand touch may activate nerve endings, distract attention, and alleviate pain by inhibiting conduction and interruption. But the stress relief, neural activation, and distraction caused by hand touch are short-term effects. This helps explain the nonsignificant sustained effect of affective touch on pain relief found. Thus, it may only be used effectively as an auxiliary means of pain relief in clinical practice. The intervention time of this study was 2 weeks, which may be too short to assess longer-term effects.

Quality of life assessment is an important part of cancer treatment and nursing effect evaluation. Quality of life is an important goal in nursing work during the limited survival period of patients with advanced cancer. Using FACT-G, this study found that affective touch intervention can effectively improve the quality of life of patients with advanced cancer, mainly reflected in the aspects of social/family status and emotional status. The intervention used in this study is similar to Zhou et al. (2017), who implemented touch interventions on elderly cancer patients, including physical and mental touch once daily for 0.5–1.0 hours each time. The reasons for intervention effectiveness may include physical touch, skin touch transmitted through the cerebral cortex, and insula network not only brings comfort, analgesia, and other effects but also promotes the secretion of oxytocin (Li et al., 2019), enhancing positive emotions and alleviating negative emotions. Physical touch is also a social behavior that can enhance social interaction. Studies (Senderovich et al., 2022) have shown that touch can effectively reduce agitated behavior and improve social functioning in patients, thus significantly improving quality of life. Concurrently, the psychological guidance of medical staff for patients’ positive and negative emotions can activate patients’ positive emotions and promote the establishment of emotional and social functions. Therefore, touch intervention achieves the effect of improving the quality of life of patients with advanced cancer, especially in the dimension of social/family and emotional status.

### Study Limitations

A single-center, small sample design was used in this study. All of the participants were recruited from one tertiary hospital in China, which is a comprehensive care hospital housing most cancer patients from the surrounding area. Thus, the sample may not be representative of patients in other areas/regions. Multicenter, large-sample studies should be implemented in the future to overcome this limitation. The participants were all patients with advanced cancer, and there were gender differences between the two groups before the intervention. Also, no detailed stratification of participants was done based on cancer type, treatment methods, treatment side effects, pain experiences, or caregiver differences. In the future, patients affected by the same disease, of the same gender, and the same treatment method may be recruited into a larger sample to improve the reliability of results and better promote the application and promotion of affective touch. Moreover, the intervention duration used in this study was short (2 weeks), and the relief of physical and mental burden was mainly attributed to the immediate impact of affective touch therapy. The sustained effects should be further explored and verified. Also, the study period should be extended in future studies to evaluate long-term efficacy, and affective touch may be compared with other psychological interventions to explore more effective and convenient interventions for patients with advanced cancer.

### Conclusions and Implications for Practice

The middle-range theory of affective touch, based on Swanson’s caring theory, successfully transforms the concept of humanistic nursing care into a simple and safe nursing intervention. Although other interventions have been shown to be effective on both physical and psychological outcomes in patients with advanced cancer, the emotional dimension of touch is a core component of the mentalization of the body’s internal environment, influencing the progressive construction of 20 multisensory, autonomic, and motor predictions of the body’s variable physiological states and how they couple with the outside world (Fotopoulou et al., 2022). The comprehensiveness of affective touch helps improve existential distress, immediate pain, and overall quality of life in patients. Therefore, affective touch is directly applicable to improving the negative physical and mental states of patients with advanced cancer. Nurses may provide a range of interventions to facilitate meaning-building processes in patients with advanced cancer. Furthermore, the realization of the meaning-building process requires not only cooperation from the nursing team but also attention to the environment surrounding the patient. In light of the dynamic interaction between the touched and the touch therapist, implementing affective touch interventions requires the cooperation of advanced nurse practitioners.
